# Contextual factors influencing the urban mobility infrastructure interventions and policies for older adults in low- and middle-income countries: a realist review

**DOI:** 10.1186/s12889-022-13875-6

**Published:** 2022-08-05

**Authors:** Divya Sussana Patil, Ajay Bailey, Uday Narayan Yadav, Sobin George, Marco Helbich, Dick Ettema, Lena Ashok

**Affiliations:** 1 Transdisciplinary Centre for Qualitative Methods, Department of Health Information, Prasanna School of Public Health, Manipal Academy of Higher Education, Manipal, India; 2 Department of Human Geography and Spatial Planning, Faculty of Geosciences, Utrecht University, Utrecht, the Netherlands; 3 National Centre for Epidemiology and Population Health, The Australian National University, Canberra, ACT Australia; 4 Centre for Primary Health Care and Equity, University of New South Wales, Sydney, Australia; 5 Centre for Study of Social Change and Development, Institute for Social and Economic Change, Bengaluru, Karnataka India; 6 MSW Program, Department of Global Health, Prasanna School of Public Health, Manipal Academy of Higher Education, Manipal, India

**Keywords:** Age-friendly cities, Contextual factors, Older adults, Low- and middle-income countries, Urban transportation, Public transport, Non-motorised transport, Transportation policies

## Abstract

Transportation is among the key aspects that influence active ageing. This realist review intends to understand the mechanisms of urban mobility infrastructure interventions and policies in low- and middle-income countries for older adults and to identify factors, which influenced the success or failure of interventions. We followed the steps suggested by Pawson and colleagues for a realist review. Electronic databases were searched from inception until August 2020. Studies were screened based on titles, abstracts and full text. The quality of included studies was assessed based on rigour and relevance. The evidence was obtained from 36 articles with diverse study designs conducted in 36 low- and middle-income countries. Findings were validated through stakeholder consultations from three low- and middle-income countries. Of the various individual factors identified, behaviour change communication interventions were low-cost, had a long-term impact and were efficient in increasing awareness among users to improve safety, social inclusion and about transport schemes for older adults. Improved transport infrastructure resulted in a shift from private to public transportation. For a sustainable urban transport infrastructure, good governance and involvement of stakeholders for planning and implementing transport interventions were considered necessary. Lack of evaluation, experience of transport planners, and inter-sectoral coordination were key challenges to successful interventions. The review highlighted a lack of older adult-specific transportation policies, and gender-targeted interventions for older women, suggesting a need for interventions and policies based on the contextual factors existing in a region.

**Supplementary Information:**

The online version contains supplementary material available at 10.1186/s12889-022-13875-6.

## Introduction

Globally, there is a rapid growth in the aging population with a simultaneous rise in the number of older adults living in cities. It is projected that between 2015 and 2050, the population of older adults above 60 years will increase from 12% to nearly 22% [1], intensifying the demand for better quality services in every sector, including urban transport. A majority of the older adults in low- and middle-income countries live in cities that are not designed according to their needs, and for them accessing essential services such as healthcare, employment and social life may involve commuting in unsafe, irregular, and expensive transport services [2–5]. The transportation needs of older adults may vary depending on factors such as employment status, possession of driver’s license, leisure activities etc. If mobility is hindered, it begins to affect their well-being [6]. The sustainable development goals, consisting of 17 goals, are a plan of action which is interlinked to achieve a sustainable future for everyone across the globe by 2030. Among them, sustainable development goals 3 and 11 are of relevance for this review. The third sustainable development goal lays emphasis on ensuring healthy lives and promoting well-being for everyone, including older adults [7]. In this context, safe and affordable mobility infrastructures are key lifelines for older adults to reduce transport-related social exclusion and improve access to essential services required for maintaining a better quality of life [8–10]. Available evidence also underscored the linkages between improved transport infrastructure and welfare outcomes of older adults. For instance, interventions such as improving transport infrastructure and urban regeneration showed an improvement in mental health and quality of life outcomes [11, 12].

Making cities sustainable by investing in public transport through urban planning and management is a part of the sustainable development goal 11 [7]. However, in low- and middle-income countries, which according to the World Bank refers to countries classified under low-income, lower-middle-income, and upper-middle-income economies [13], the urban residents, especially older adults, face difficulties in accessing transportation that limits their contributions to the society and the ability to lead productive lives [3, 14]. In recent years, to cope with rapid urbanisation, a few initiatives towards improving access and safety in urban transport infrastructure have been undertaken for older adults in different low- and middle-income countries [15–17]. Despite efforts from national and international agencies to address the needs of older adults concerning public transportation (for e.g., bus rapid transit systems, bus corridors with priority lanes, improvement in public transport and non-motorised transport services) inequalities faced by older adults while accessing urban transport infrastructure are noticeable across regions [14, 18, 19]. Subsidised fare made public transport affordable to the most vulnerable population in Latin American countries such as Columbia and Chile [20]. Similar interventions can be adapted in other low- and middle-income countries to make public transport accessible to older adults irrespective of their income levels.

It is evident that in low- and middle-income countries, implementation of interventions is limited by context-specific social, economic, and political barriers [21, 22]. In this light, comprehensive scientific information on the underlying mechanisms of why an intervention was successful or not to ensure equitable access for older adults to mobility infrastructure remained unexplored. A deeper understanding of intervention mechanisms in the context of low- and middle-income countries can be provided through a theory-driven and interpretive approach, i.e., a realist review given by Pawson and colleagues (2005) [23]. Although this method has not been used previously to synthesise evidence in the area of transportation interventions, it has the potential to provide comprehensive evidence to guide policy and practice in this area of research.

A realist review encapsulates existing theory, evidence from previous research and stakeholder consultations in understanding the issues around the implementation of interventions with a contextual lens. Realist reviews have been used in understanding complex interventions related to health, housing, education etc. [23, 24]. Therefore, this realist review could help us identify the gaps in researched transportation interventions and the generated findings could guide policymakers to design projects or re-design the existing mobility services. The following research questions will be addressed through this review 1) what are the urban mobility infrastructure interventions and policies/policy measures in low- and middle-income countries to improve the accessibility and safety of older adults? (2) How do contextual factors influence the success or failure of such interventions and policies/policy measures?

For this review, we apply the following definitions:

1. Mobility refers to “movement in all of its forms, including basic ambulation, transferring from a bed to a chair, walking for leisure and the completion of daily tasks, engaging in activities associated with work and play, exercising, driving a car, and using various forms of public transport” [25].
2. For the purpose of this research, ‘Urban mobility infrastructures’ refers to infrastructures (physical infrastructures in the cities) and services (e.g., public transport services running on the infrastructures), as well as non-motorized transport (i.e., cycling and walking).
3. Transportation interventions in this research refer to improvements made in public transportation and non-motorized transportation services and infrastructure, and the legal and economic policies outlined by the government with respect to transportation.
4. Stakeholders for the current research include transport policymakers, transport intervention implementers, transport planners, urban planners (government and private), transportation experts, non-government organization, researchers, and older adults from low- and middle-income countries.
5. “Well-being includes the presence of positive emotions and moods (e.g., contentment, happiness), the absence of negative emotions (e.g., depression, anxiety), satisfaction with life, fulfilment and positive functioning” [26].

## Methodology

The protocol for this review was registered with the PROSPERO database (registration number: CRD42020168020). The realistic review involved seven phases explained below.

### Scoping the literature

**Fig. 1:**
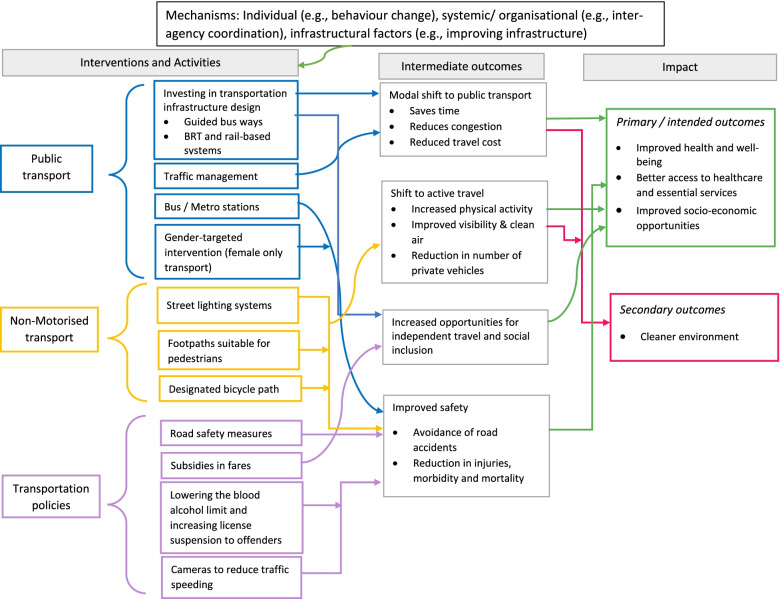
Program theory explaining the pathway of transportation interventions and policies and its outcomes

An initial program theory was developed after identifying the interventions and policy measures/policies through preliminary literature search, and discussion with stakeholders. The program theory represented in Fig. 1 shows how the implementation of transport interventions and policies influenced the outcomes such as improved health and well-being, better access to healthcare, social life and employment, and cleaner environment. The transport interventions were categorised as public transport interventions, non-motorized transport interventions, and transportation policies, which were influenced by various mechanisms contributing to their success or failure. This program theory further helped inform the eligibility criteria and to develop the search strategy for a systematic search.

### Search process

A systematic and comprehensive search for empirical evidence was conducted on electronic databases such as Scopus, PubMed, ProQuest, Web of Science, EMBASE, JSTOR, Organisation for Economic Co-operation and Development library, Mobility in Cities, Transport Research International Documentation, World Health Organization-Institutional Repository for Information Sharing, International Initiative for Impact Evaluation, Sustainable Urban Transport Project, and Google Scholar from inception until August 2020. The search strategies and keywords used are given in additional file 1. Additional literature from reference lists of included studies, relevant review reports, conference proceedings, doctoral theses, and dissertations, were examined for potential studies. Search was not restricted to publication type but was limited to studies on humans and published in English language.

### Selection and appraisal of documents

#### Study design

We included all relevant studies such as quantitative, qualitative, mixed methods studies, literature reviews, reports, theses, website articles, secondary data research and systematic reviews that dealt with the issue of improving the safety and accessibility of urban mobility infrastructure.

#### Population

We included all studies that informed about urban mobility infrastructure interventions and policies/policy measures in low- and middle-income countries for older adults and all users (literature that included older adults as subgroup).

#### Outcome

We included those studies that focused on the impact of urban transport infrastructure interventions and policies/policy measures on socio-economic opportunities, well-being, and health. Studies that did not give sufficient information about the underlying mechanisms of an intervention were excluded. Studies that focused only on specific age groups apart from older adults were excluded. Data was managed using Zotero software [27].

Screening was undertaken based on title and abstract initially by DSP and further it was assessed by UNY. This was followed by a full text screening initially by one reviewer and assessed by a second reviewer. Any disagreements were resolved by discussion. The reasons for exclusion have been recorded. Quality assessment was done by DSP and subsequently cross checked by UNY. The appraisal was based on the relevance and rigor of a study, which is followed for a realist review. We considered a study relevant if the data contributed in building the theory, and rigor of the study was assessed after discussion within the review team regarding the methods used to generate data. Only those studies which established both rigor and relevance were included in the review.

### Data extraction

Data extraction sheet was pilot tested initially on few articles; subsequently the data extraction sheet was improved further before proceeding with data extraction. Data were extracted by DSP and double-checked by UNY. Any disagreements were resolved by discussion. The data extraction sheet included details of study characteristics, intervention related information, details of the program theory and quality of the study.

### Analysis and synthesis process

The results are presented as per the *Realist and MEta-narrative Evidence Syntheses: Evolving Standards* reporting guidelines (additional file 2) [28]. The mechanisms that influenced the success and failure of interventions and transportation policies were synthesised and presented narratively.

### Stakeholder consultation and refining the initial program theory

Stakeholder inputs are necessary for policymaking and are recommended for a realist synthesis [23]. Stakeholders were consulted before starting the review, during the review process and after completing the review. During the review process, we consulted stakeholders (*n* = 12) from India, Bangladesh, and Nepal, to understand their perspectives regarding urban mobility infrastructure interventions. After sharing the initial review findings with them, online consultations were conducted. The discussion was recorded after obtaining consent and later transcribed. The discussion involved their opinions about urban mobility infrastructure interventions and validation of findings from the review. Similarly, another online stakeholder consultation event was held to disseminate the findings, which was attended by stakeholders (*n* = 17) from India, Bangladesh, the United States of America, Norway and Indonesia. The information was used to refine the program theory (Fig. 1).

### Changes in the review process

Studies focusing only on transport interventions and policies for older adults were limited. Hence, we used a broader search strategy and included studies that mentioned ‘all users’. Any study that mentioned ‘all users’ but did not include older adults was excluded.

## Results

**Fig. 2:**
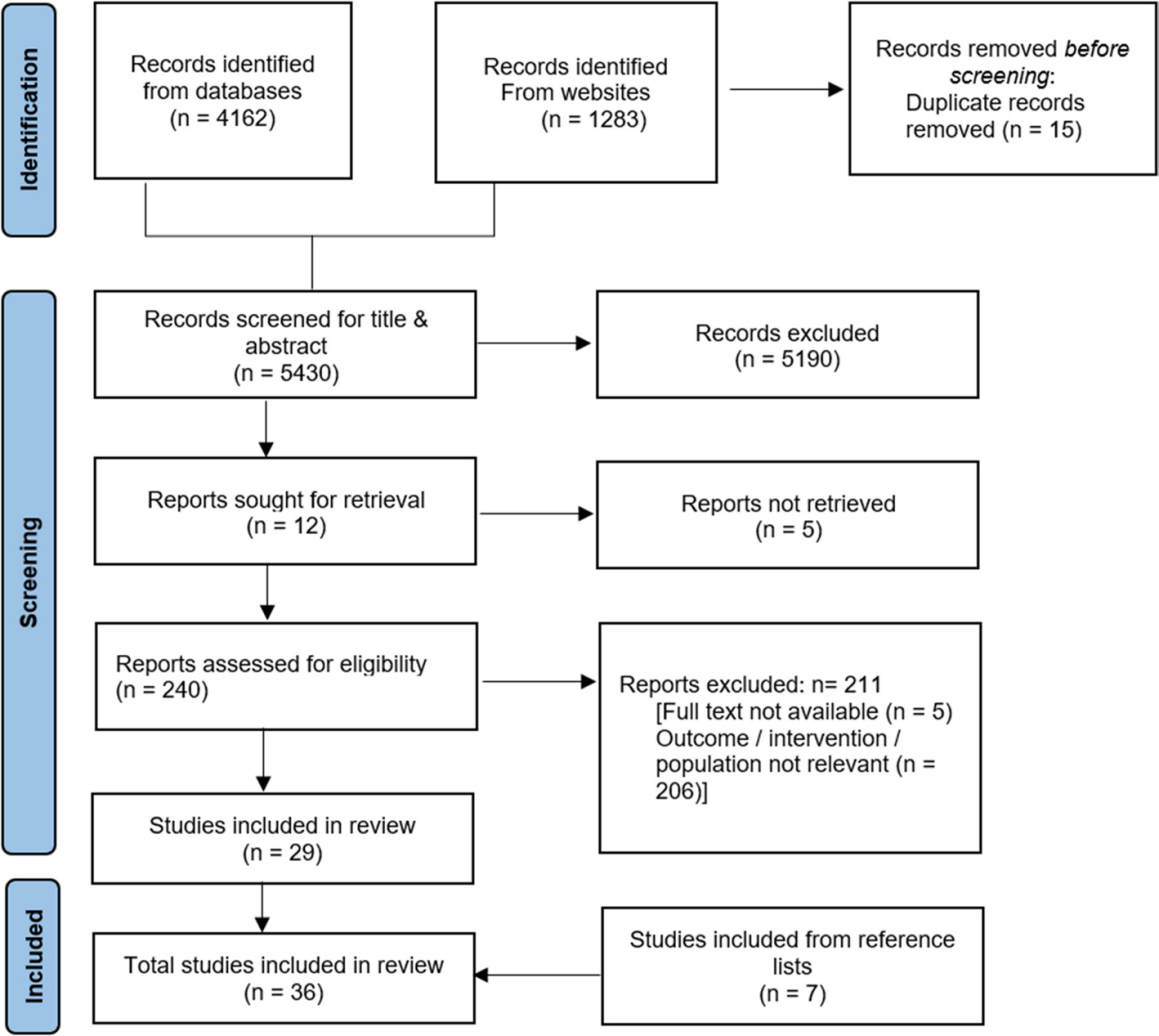
Document flow diagram. *Adapted from: [29]

A total of 36 studies met the inclusion criteria, of which 16 focused on urban mobility infrastructure interventions, and 20 were related to transport policy/ policy measures. The study selection process is shown in Fig. 2. Evidence was obtained from literature reviews (*n* = 18), systematic reviews (*n* = 3), reports (*n* = 8), survey (*n* = 2), case studies (*n* = 2), randomised controlled trial (*n* = 1), mixed methods (*n* = 1), and qualitative content analysis (*n* = 1). Evidence came from 5 low-income countries, 18 lower-middle-income countries, and 13 upper-middle-income countries. The characteristics of included studies are given in detail in table 1a and 1b (additional file 3).

### Main findings

**Fig. 3:**
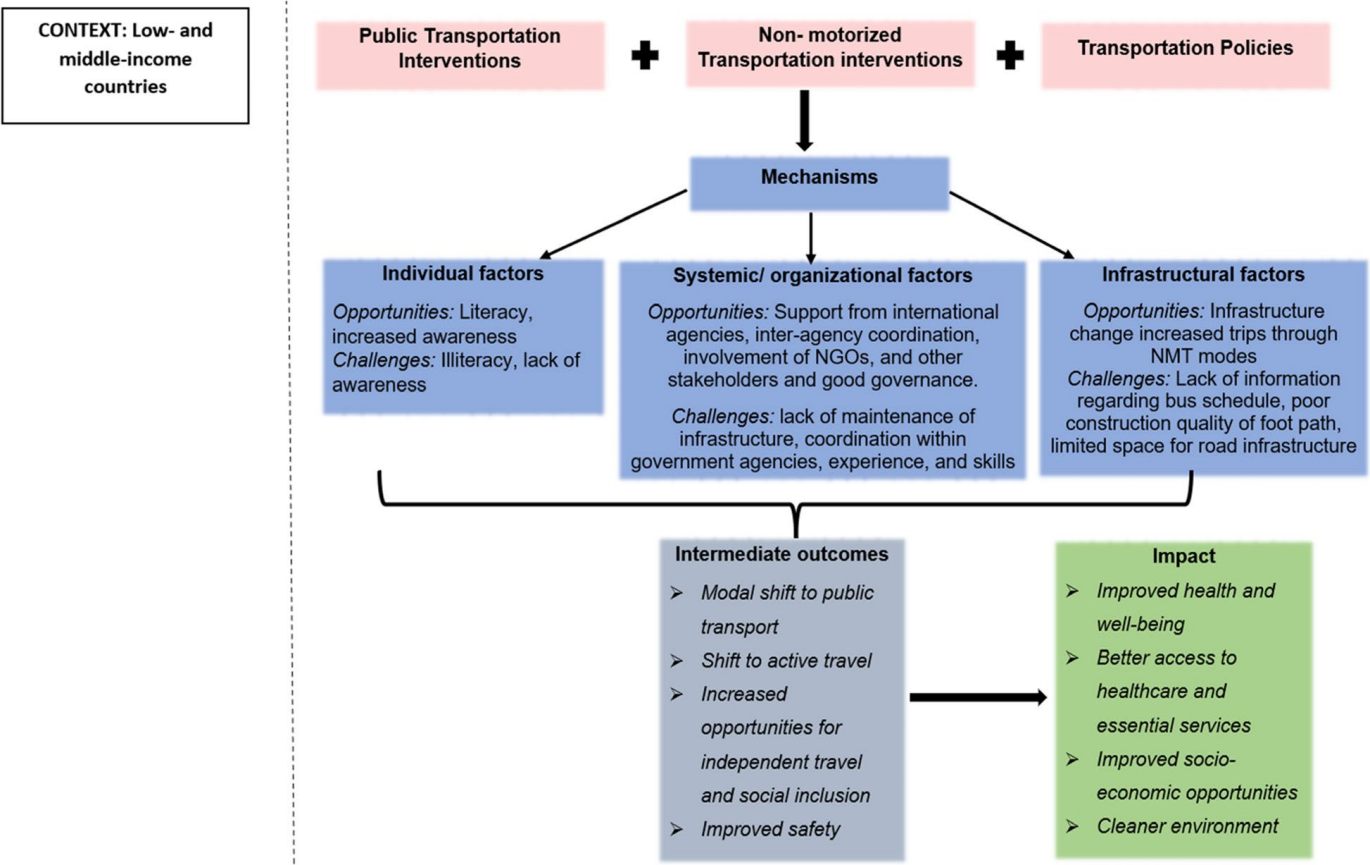
Mechanisms that influenced transportation interventions and policies

The review identified various transport interventions and policies/ policy measures with respect to public transport and non-motorised transport, which focused on improving infrastructure, safety, air quality, access to health care, and employment and social services in low- and middle-income countries. Figure 3 summarises the interventions identified with respect to public transport, non-motorised transport, and transport policies, the mechanisms that influenced them and the expected outcomes. The mechanisms such as individual factors are attributed to older adults’ behaviour, literacy levels, awareness about policies etc. Systemic/ organisational factors include the government agencies at local, state and country levels, non-government organisations, and stakeholders involved in transport interventions. In this review, infrastructural factors refer to the interaction between the systems and organisations since they overlap. For example, bus timing information and maintenance of infrastructure are services offered by the transport organisations.

### Interventions and policies focused on older adults in low- and middle-income countries

The review identified three transport interventions and one policy, specifically focusing on older adults. This indicates that majority of the low- and middle-income countries do not plan transport policies and interventions explicitly for older adults. Interventions such as placing stickers with evocative messages (with or without images) inside the ‘Matatus’ or ‘mini buses’ along with a radio campaign in Kenya empowered older adults to complain about rash driving, thus reducing accidents and having a safe commute [30]. Likewise, ‘Boda-boda’ (motorcycle taxis) in Nigeria and South Africa helped older adults get access to remote areas, which helped them avail timely healthcare services and improved access to labour markets to sell their produce [31]. In Mexico, the pedestrian program *‘Camina’* showed increased shift to active transport among older adults [32]. In addition, stakeholders’ consultation revealed that countries such as Nepal and India had policies for fare concession and reserved seats for older adults in buses. However, few older adults were unaware of these policies. Hence, pamphlet distributions in public places helped increase awareness among older adults. Tables 2a, 2b and 2c (additional file 4) give a detailed context-mechanism-outcome configuration of transport interventions and policies for all users, which included older adults as well.

### Mechanisms that influenced transportation interventions in low- and middle-income countries

#### Individual factors

The behaviour change communication interventions such as using stickers with messages, messages aired through radio and distributing pamphlets with messages to increase awareness about safety while travelling in public transport [30] were successful and influenced by individual factors such as literacy and awareness about transport services. This when combined with simultaneous improvement in public transport (e.g., increased frequency, electronic ticket system, condition of buses/ bus stops) and non-motorised transport infrastructure (e.g., clean sidewalks, streetlights) resulted in behaviour change among older adults. The reviewed studies in countries like Mexico, African cities, India, Columbia, China, and Morocco showed a change in behaviour such as increased walking for transport and increased use of public transport among users (including older adults) once their requirements regarding transport infrastructure were met [30, 32–39]. Similarly, to promote the ‘seat offering culture’ in public transport, a short film was disseminated in Wan Chai district, China, through social media platforms, holding tram parades and talks in schools. This showed improved social inclusion of older adults, respect and age-friendliness in transportation [40].

On the other hand, illiteracy was seen as a barrier to the success of interventions, where stickers or pamphlets were used to educate people [30]. Similar observation was made in China, where public information campaigns to create awareness about pro-environmental travel behaviour helped in increasing the use of public transport and walking but did not have a sustainable long-term impact. This was attributed to the lack of knowledge about transport services, a prerequisite for change in behaviours [19].

Stakeholder consultation during the review revealed that distribution of pamphlets and stickers in public areas helped increase awareness among Nepal's public about reserved seats for older adults. Transport personnel helped older adults get seats in the bus and gave sufficient time for older adults to board and alight from the bus. However, emphasis was placed on the lack of awareness among older adults in Nepal about the current schemes, such as concessions in bus fares and designated seats for older adults.

#### Systemic and organisational factors

Transportation planning and policy demand good governance for the use of existing resources and for allocating new resources. Our results highlight that successful interventions for older adults to safely access public transport mainly weighed on inter-agency coordination (local and state governments), adequate funding from respective departments, proper planning, implementation and monitoring [36], strong commitment from the local government, and stakeholder involvement. For example, China involved multiple bus agencies in providing cost effective bus services. The systemic and organisational factors mentioned above led to the successful implementation of bus rapid transit in China [32, 36, 38]. Similarly, the government’s decision to expand telecommunication services alongside the introduction of *Boda-Boda* (motorcycle taxis) facilitated mobility and access to healthcare and markets for older adults in Nigeria and South Africa [31].

However, a lack of trained personnel, corruption, limited experience in transport planning and implementation, and lack of awareness about the needs of older adults to access transport infrastructure were barriers to successful interventions. For instance, the MyCiti project in South Africa identified municipal capacities, operational difficulties, teamwork, ethics, and geographical spread as challenges for project implementation [41]. In Pakistan, although there were several policy measures planned to improve transport infrastructure and to have an efficient transport system the above mentioned factors acted as barriers for the successful implementation [42, 43].

Consultation with stakeholders in Nepal indicated that, the senior citizen’s act 2063 (2006) was passed by the Nepal law commission due to the successful advocacy by non-government organisations for fare reduction by 50% and dedicated seats for older adults in public vehicles [44]. The other enabling factors identified during stakeholder consultations were efficient management of metro rail in Delhi, India and public consultation during the feasibility stage for bus rapid transit and mass rapid transit in Dhaka, Bangladesh. However, the stakeholders also highlighted that lack of inter-agency coordination, funding, absence of user-oriented thinking, lack of long-term outlook while planning interventions, lack of knowledge for the need to invest in low-cost solutions such as bus and non-motorised transport, and prioritising private vehicles were key challenges for a successful intervention. In countries like India, as mentioned by one of the stakeholders, lack of evaluation of the projects was an important factor for the failure of interventions. For example, the metro projects were beneficial in bigger cities like Delhi; however, planning for the metro without a proper feasibility check resulted in massive failure of metro projects in a few regions similar to the Jaipur metro project [45].

#### Infrastructure-related factors

Improving the design of public transport and non-motorised transport, having good first mile and last-mile connectivity, bus rapid transit or bus priority lanes encouraged users to shift the mode of transport from private to public. In India and Mexico, introducing the electronic ticketing system, comfortable seating arrangements in the buses and bus stations, proper bus shelters and exclusive bus lanes helped in reducing travel times and encouraged the older adults to use buses [35, 37]. A study conducted in the United States of America, United Kingdom and Hong Kong showed similar findings, where older adults and persons with disabilities were more likely to use bus services when the design of the bus and bus stop improved [46]. However, an unfriendly bus design, no concession in bus fare, and lack of designated seats for older adults pushed them to opt for other modes of transport.

Stakeholder consultation from India revealed that bus rapid transit was successful in various Indian cities like Ahmedabad, Surat, and Hubli-Dharwad due to the design of the infrastructure. It was suggested that bus rapid transit was successful mainly in medium-sized cities, with good last-mile connectivity and short average trip lengths. The success of a bus rapid transit depended on the selection and design of the corridor. The public bike-sharing project implemented in Mysore, India was another success story due to the dedicated lanes for cyclists and planned parking spaces. In Bangladesh, new footpaths are being constructed, which are designed in a way that is friendly for older adults as well as for people with disabilities. In Dhaka, Bangladesh, the lack of older adult friendly buses and dedicated seats, crowded buses and no concession in bus fares have pushed the older adults to use the non-motorised rickshaws. Additionally, the non-motorised rickshaws provide door-to-door delivery; hence, older adults preferred using them to the buses.

##### Cultural context

In Bangladesh, one of the stakeholders mentioned that there are no dedicated seats for older adults in the bus; however, there is a cultural element about respecting older adults, hence when an older adult enters the bus the younger people tend to offer them their seat. This cultural context is often seen in South Asian countries. In such regions, behavior change communication interventions can be effective in educating the younger generation to be more sensitive to the transportation needs of older adults.

### Transportation policies and policy measures in low- and middle-income countries

#### Road safety measures

Cities having a good rail transit network, road and vehicle infrastructure, bicycle tracks, had lower rates of road traffic injuries [47–51]. Various transportation programmes initiated in regions across Asia, South America, the former Soviet Union and low-income African countries, did not focus on reducing motor vehicle use by improving non-motorised transport design. Furthermore, there was a lack of investment in safer public or active transport alternatives other than cars, which are not affordable to everyone [48]. Policies that aimed at the construction of new infrastructure to separate the pedestrians from motor vehicles put vulnerable road users at higher risk of crime, and jaywalking put the pedestrians at greater risk [48]. Despite extensive research conducted in Pakistan on the importance of transport policies and their effect on health, there have been no interventions suggested for betterment. This indicates a need for concentrated efforts by the government and society to implement necessary actions [52]. The review identified that lack of legal monitoring framework, unplanned urbanisation, absence of suitable infrastructure, and a surge in motorisation as challenges to improving road safety [51]. In order to determine new interventions to improve safety, there is a need for reliable accident data, but we identified a lack of reliable accident data in Asian countries due to under-reporting, unlike the high-income countries where good progress is made in the area of road traffic safety due to capacity building, research and development [51]. Therefore, we recommend that low- and middle-income countries have a more robust system for recording accident data in order to design new policies as per requirement.

#### Thrust on access to healthcare, employment, and leisure

The review highlighted that subsidy in transport fare resulted in better employment opportunities in South Africa [53] and various transportation projects in Kenya, Uganda and Ecuador helped improve access to healthcare and essential services [54]. Similar findings were observed in Seoul, South Korea where the introduction of a fare-free subway policy for older adults resulted in increased use of public transit than other transit modes [55]. Likewise, evidence from the United Kingdom shows that the free bus pass scheme helped in increasing physical activity and reducing social exclusion by giving older adults a sense of belonging in the community [56]. Older adults in low- and middle-income countries may have varying degrees of financial hardships. Hence, such initiatives can be planned and implemented in low- and middle-income countries to increase the opportunities for older adults to access healthcare, work and leisure activities.

#### Policy measures for improved environmental conditions and well-being

Energy-efficient transport systems can be promoted by developing mass rapid transit, upgrading the public transport (bus/tram), developing safe pedestrian and bicycle lanes and encouraging the use of small and highly efficient vehicles [52]. Evidence from low- and middle-income countries showed that low carbon emissions combined with active travel policies (for e.g., promotional campaigns, change in physical infrastructure focused on safety, reduction in vehicle speed etc.) helped in reduction of number of years of life lost due to ischemic heart disease, improved the health of individuals and a cleaner environment [57, 58]. A shift to an active mode of transportation has shown health benefits among older adults such as reduced cardiovascular diseases in Canada and other European countries. In addition, non-motorised transport has helped reduce emissions of harmful gases, reduced noise pollution and made neighbourhoods liveable for the residents [59–62]. Therefore, it is important to promote active transport and transportation modes that are energy efficient to have a cleaner environment, which further enhances the well-being of older adults. A multi-sectoral approach such as involving transport planners, public health, and environment experts is recommended while planning and implementing interventions.

#### Measures to improve non-motorised transport

Inadequate implementation of non-motorised transport specific policies in African countries was attributed to lack of an action plan, research and development, and monitoring and evaluation [63]. In addition, budget allocation and lack of adequate space were other issues faced by low- and middle-income countries [64, 65]. On a positive note, under the sub-Saharan Africa transport policy programme, Nairobi successfully implemented interventions such as traffic calming measures, supply of bicycles, and building of special infrastructure for cyclists and pedestrians. In recent years, non-motorised transport has been gaining popularity due to both health and environmental benefits. However, the number of trips made using non-motorised transport is dependent on the quality of pedestrian pathways, and bicycle lanes [66]. Therefore, it is important for the transport sector to look into the specific needs of older adults and design suitable infrastructure accordingly.

## Discussion

This realist review summarised the findings of 36 articles in 36 low- and middle-income countries on various urban transport infrastructure interventions, why certain interventions and policy measures were successful or not successful. It is important to identify and make note of failures in the current interventions and policy measures, which restrain further progress. At the same time understanding the reasons for successful interventions will help policymakers and transport planners design interventions according to the context. Sustainable transport infrastructure, which promotes social inclusion and safety will eventually have an impact on the well-being of older adults [67, 68]. Some of the key observations from our review are discussed below.

Behaviour change communication interventions are affordable and have a better prospect at long term sustainability. It has been used effectively in various public health and transport interventions to obtain desired outcomes [69–73]. For example, educational campaigns for users through information kiosks in Brisbane and using informative models of the stations and vehicles in Peru before implementation of the project resulted in better public acceptance of public transport [43]. In the context of this review, it was identified that educational interventions alongside an improvement in transport infrastructure helped in better acceptance of public transport followed by a shift from private to public and motorised to non-motorised transport. It was observed that an increase in public transport users simultaneously increased active transportation to access the public transport transit stations [62, 74]. Evidence from developed countries showed that an improvement in transport infrastructure had subsequently increased walking for transport, which helped in promoting physical activity for older adults resulting in their better well-being [75–79]. Likewise, empowering older adults to voice their concerns helped reduce road accidents and thus accident-related injuries and fatalities due to rash driving. Additionally, sensitizing the public and transport personnel helped them to be more considerate towards older people, further improving social inclusion of older adults in public transport. However, considering the fact that illiteracy was a barrier to behaviour change communication interventions, more images rather than text messages could be used while communicating information.

Inadequate funding in various low- and middle-income countries acted as a barrier to the implementation of inclusive transportation interventions. In such situations, it is beneficial to identify low-cost solutions to provide sustainable transport options. Public–private partnership in transport interventions in China where multiple bus agencies were involved in bus rapid transit was found to be cost-effective. Similarly, public–private partnership initiatives in a few cities of developing countries like Latin America and Asia have shown significant benefits in urban public transport, especially with respect to developing socially inclusive communities [80, 81]. However, a few challenges with respect to public and private agencies were seen in Maharashtra, India, where public–private partnership initiatives have been taken up [82]. Therefore, we recommend that contextual factors at systemic/ organization level should be considered before taking up public private partnership initiatives in other low- and middle-income countries [83].

The review brought to light that involvement of stakeholders during the projects’ design phase was lacking. A case study from the city of Indore, India reported that the importance given to public outreach by the implementing authority contributed to the success of bus rapid transit (iBus) in Indore [14]. Hence, it is recommended that public consultation should be given importance before planning for projects. Another important issue, which was highlighted during the stakeholder consultation was the lack of proper evaluation after the implementation of the project. New projects were planned even before evaluating the previous project. Though bus rapid transit is a low-cost project, suitable for medium-sized cities, there is a [perhaps wrong] notion by the authorities that it is not feasible for countries like India and Bangladesh. This suggests the importance of investing in low-cost solutions like bus rapid transit and non-motorised transport infrastructure. Hence, a detailed evaluation of implemented projects before drawing conclusions is strongly recommended for all transport infrastructure interventions.

It was evident from the review as well as stakeholder consultations that policy initiatives were available for older adults, but the implementation of such policies are not up to the mark. Therefore, many interventions to improve infrastructure are not sensitive to the needs of older adults. There are universal accessibility guidelines for all vulnerable groups, which may miss out few minor challenges faced by older adults. According to the age-friendly cities guidelines, transportation is one of the important domains affecting the well-being of older adults [84]. Research emphasising the importance of transport policies and health outcomes has been conducted, but a lack of commitment from the government in engaging the community to design and implement interventions was observed in a few low- and middle-income countries. A few stakeholders in our research highlighted the importance of gender-specific targeted interventions for older women. Older women felt unsafe while travelling on the public transport and walking alone after it was dark due to thefts, inadequate street lighting, lonely streets, and inappropriate behaviour of men on the bus, for example sitting in the seats allotted for women or standing closer to where women were sitting [85, 86]. Additionally, they faced challenges with the poorly maintained infrastructure of buses as well as walking pathways due to frailty. It is reported that older women are frailer and have much higher incidents of falls compared to older men [87–89]. However, the review failed to identify such interventions in low- and middle-income countries specifically designed for older women, thus suggesting the need to focus on transport interventions for older women. Policymakers could use recommendations from available research, to design transport infrastructure with a focus on older women.

### Strengths and limitations

The strength of this realist review is that it helped us uncover the reasons for desired or undesired outcomes of certain interventions, which were validated through the valuable insights from stakeholders. The stakeholders were from government, private, non-government organizations, and older adults, which gave a wide range of opinions. Using a theory-driven method and systematic search of literature helped us in understanding the key processes for successful interventions and policy measures. Transport planners, urban planners, policymakers and relevant stakeholders can use these findings for future urban transport interventions and policies.

The limitations of the study were as follows; First, there was a lack of information regarding details of evaluation of interventions in most of the studies. This informs that future research has to focus on conducting a robust evaluation of transport interventions. Second, the limited literature on interventions and policy measures for only older adults resulted in modifying the search strategy to ‘all users’, suggesting the need to further explore in detail the mobility requirements of older adults and how policymakers can improvise transport interventions to make transport infrastructure inclusive for older adults. Third, stakeholders’ consultation was limited to a few low- and middle-income countries only due to the inability to get a response from those whom we contacted (due to the pandemic) and time constraints, but this limitation was overcome by contacting stakeholders who had rich experience of working on different transport projects in the low- and middle-income countries.

## Conclusion and recommendations

An important implication for research is that there is a need of an implementation and evaluation plan for the interventions and policy measures in place. More importantly, local authors should use the evidence from previous interventions/programs to design and implement the next one, or else the taxpayer’s money will not be well recognised. To make the older adults feel included in a society it is important to re-think transportation policies and re-design the existing transport infrastructure to suit their mobility needs. An integrated approach is necessary in building age-friendly communities, which will improve the overall well-being of older adults. Based on our findings, behaviour change communication approach was effective to increase awareness among individuals and improving the safety of users by reduction of road traffic injuries. Efforts to collaborate with various stakeholders such as local administration, schools, community members and non-government organizations are important to make the behaviour change communication approach effective. Improving public transport and non-motorised transport infrastructure led to a shift from private to public mode of transport, which has a long-term impact on the well-being of individuals. Therefore, ensuring good governance, coordination between departments, skilled personnel, adequate funding for project sustainability, and stakeholder consultation before planning any intervention will result in developing sustainable, cost effective, and socially inclusive urban transport infrastructure.

## Supplementary Information

**Additional file 1:** **Part A: Search keywords. **The keywords were modified for different databases as required. **Part B.** Search strategy and results.

**Additional file 2.** Table with RAMESES checklist.

**Additional file 3:** **Table 1a. **Study characteristics- Transportation interventions. **Table 1b.** Study characteristics- Transport policies and policy measures.

**Additional file 4:** **Table 2a.** Urban transport interventions (public transport). **Table 2b.** Urban transport interventions (non-motorised transport).

## Data Availability

Data sharing is not applicable to this article as no datasets were generated or analysed during the current study.
