# Supplementary material for: Contextual factors influencing the urban mobility infrastructure interventions and policies for older adults in low- and middle-income countries: a realist review
Source: BMC Public Health. 2022 Aug 5;22:1489. doi: 10.1186/s12889-022-13875-6 (PMC9354419; doi:10.1186/s12889-022-13875-6)
Supplement: Supplementary file 1 — Additional file 1: Part A: Search keywords. The keywords were modified for different databases as required. Part B. Search strategy and results. [file 12889_2022_13875_MOESM1_ESM.docx]

**Part A: Search keywords:** The keywords were modified for different databases as required

| **Population**  “old* people” OR “old* persons” OR “older adults” OR “elder*” OR “elder* persons” OR “aged people” OR “aged population” OR “aging population” OR “ageing” OR “aging” |
| --- |
| **Intervention**  “Transport* infrastructure” OR “mobility infrastructure” OR “transport* intervention” OR “public transport” OR “public transit” OR “urban mobility infrastructure” OR “Motorized transport” OR “Non-motorized transport” OR “transport* planning” OR “transport* design” OR “modes of transport” OR “means of transport” OR “a form of transportation” OR “electric motor” OR “road transport” OR “roadways vehicle” OR “municipal transport system” OR “metropolitan transport system” OR “municipal mobility” |
| **Outcome**  “Well-being” OR “improved transport*” OR “quality of life” OR “health” |

**Part B: Search strategy and results:**

| **Database** | **Search strategy** | **Results** |
| --- | --- | --- |
| PubMed | "public transport" OR "Public transportation" OR "transport infrastructure" OR "mode of transport" OR "public transport interventions" OR "urban infrastructure interventions" Filters: Humans, English, Middle Aged: 45-64 years, Aged: 65+ years, 80 and over: 80+ years, Middle Aged + Aged: 45+ years, from 1000/1/1 - 2020/6/30 | 4843 (first 421 downloaded) |
| PubMed | "urban transportation infrastructure" AND health Filters: English, from 1000/1/1 - 2020/6/30 | 320 |
| Scopus | ( "Transport* infrastructure" OR "mobility infrastructure" OR "transport* intervention" OR "public transport" OR "public transit" OR "urban mobility infrastructure" OR "Motorized transport" OR "Non-motorized transport" OR "transport* planning" OR "transport* design" OR "modes of transport" OR "means of transport" OR "a form of transportation" OR "electric motor" OR "road transport" OR "roadways vehicle" OR "municipal transport system" OR "metropolitan transport system" OR "municipal mobility" ) AND ( "old* people" OR "old* persons" OR "older adults" OR "elder*" OR "elder* persons" OR "aged people" OR "aged population" OR "aging population" OR "ageing" OR "aging" OR "vulnerable population" OR "Vulnerable persons" ) AND ( "Well-being" OR "improved transport*" OR "quality of life" ) | 2365 |
| Web of Science | TOPIC: (“old* people” OR “old* persons” OR “older adults” OR “elder*” OR “elder* persons” OR “aged people” OR “aged population” OR “aging population” OR “ageing” OR “aging” OR “vulnerable population” OR “Vulnerable persons”)  Indexes=SCI-EXPANDED, SSCI, A&HCI, CPCI-S, CPCI-SSH Timespan=1988-2020  TOPIC: (“Transport* infrastructure” OR “mobility infrastructure” OR “transport* intervention” OR “public transport” OR “public transit” OR “urban mobility infrastructure” OR “Motorized transport” OR “Non-motorized transport” OR “transport* planning” OR “transport* design” OR “modes of transport” OR “means of transport” OR “a form of transportation” OR “electric motor” OR “road transport” OR “roadways vehicle” OR “municipal transport system” OR “metropolitan transport system” OR “municipal mobility”)  Indexes=SCI-EXPANDED, SSCI, A&HCI, CPCI-S, CPCI-SSH Timespan=1988-2020  TOPIC: (“Well-being” OR “improved transport*” OR “quality of life”)  Indexes=SCI-EXPANDED, SSCI, A&HCI, CPCI-S, CPCI-SSH Timespan=1988-2020  #3 AND #2 AND #1  Indexes=SCI-EXPANDED, SSCI, A&HCI, CPCI-S, CPCI-SSH Timespan=1988-2020 | 65 |
| Transport Research International Documentation (TRID) | public transport interventions | 904 |
|  | public transport interventions for older adults | 10 |
|  | safety of older adults in public transport | 156 |
| ScienceDirect  ProQuest | (“old* people” OR “old* persons” OR “older adults” OR “elder*” OR “elder* persons” OR “aged people” OR “aged population” OR “aging population” OR “ageing” OR “aging” OR “vulnerable population” OR “Vulnerable persons”) AND (“Transport* infrastructure” OR “mobility infrastructure” OR “transport* intervention” OR “public transport” OR “public transit” OR “urban mobility infrastructure” OR “Motorized transport” OR “Non-motorized transport” OR “transport* planning” OR “transport* design” OR “modes of transport” OR “means of transport” OR “a form of transportation” OR “electric motor” OR “road transport” OR “roadways vehicle” OR “municipal transport system” OR “metropolitan transport system” OR “municipal mobility”) AND (“Well-being” OR “improved transport*” OR “quality of life” )Limits applied | 884 |
| Mobility in cities database | Reports/policies | 35 |
| Google scholar | Urban infrastructure, transport infrastructure, public transportation, sidewalks, transport interventions, transport policies, pedestrian path, urban transportation, bus, metro, train, motorized transport, non-motorized transport, well-being, health outcomes | First 60 studies were considered |
| EMBASE | “old* people” OR “old* persons” OR “older adults” OR “elder*” OR “elder* persons” OR “aged people” OR “aged population” OR “aging population” OR “ageing” OR “aging” AND “Transport* infrastructure” OR “mobility infrastructure” OR “transport* intervention” OR “public transport” OR “public transit” OR “urban mobility infrastructure” OR “Motorized transport” OR “Non-motorized transport” OR “transport* planning” OR “transport* design” OR “modes of transport” OR “means of transport” OR “a form of transportation” OR “electric motor” OR “road transport” OR “roadways vehicle” OR “municipal transport system” OR “metropolitan transport system” OR “municipal mobility” AND “Well-being” OR “improved transport*” OR “quality of life” OR “health” | 47 |
| SUTP | Searched all publication in their list | 132 |
| WHO-Iris | Searched all publication in their list | 6 |
| 3IE | Searched all publication in their list | 15 |
| JSTAR – Japanese study of aging and retirement | older adults and transport infrastructure | 5 |
| OECD | Transport | 20 |
