# Supplementary material for: Contextual factors influencing the urban mobility infrastructure interventions and policies for older adults in low- and middle-income countries: a realist review
Source: BMC Public Health. 2022 Aug 5;22:1489. doi: 10.1186/s12889-022-13875-6 (PMC9354419; doi:10.1186/s12889-022-13875-6)
Supplement: Supplementary file 3 — Additional file 3: Table 1a. Study characteristics- Transportation interventions. Table 1b. Study characteristics- Transport policies and policy measures. [file 12889_2022_13875_MOESM3_ESM.docx]

**Table 1a: Study characteristics- Transportation interventions**

| Sl.No | Title | Author & year | Country | Study design | Study objective / intervention | Results |
| --- | --- | --- | --- | --- | --- | --- |
| 1 | Impact of new rapid transit on physical activity: A meta-analysis. | Hirsch et al., 2018 | Mexico | Systematic review | Impact of bus rapid transit (BRT) and Complete Street implementation on walking and cycling levels | There was an increase in walking for transport and recreation, and improved pedestrian accessibility and safety due to redesigning of streets. No increase in cycling for transport, suggesting that improvement in cycling infrastructure was not enough to make cycling an attractive, safe and convenient mobility option. |
| 2 | Mobility, transport and older people's well-being in sub-Saharan Africa: Review and prospect | Porter et al., 2018 | Sub-Saharan Africa | Mixed methods | To understand older people’s mobility and transport needs, which has been conducted to date in urban and rural contexts across Anglophone countries in sub-Saharan Africa (Kibaha research project). | Motor cycle taxi’s (Boda-Boda) offered a major employment opportunity. Small-scale farming among many older people made Boda-boda feasible to transport farm produce and farm inputs. Door-to-door services and availability at any time was an advantage. Additionally, cell phone expansion in Tanzania as across much of Africa, despite user challenges (airtime cost, network, charging) has helped older adults to use Boda-boda services |
| 3 | The Role of Quality Assessment for Development of Sustainable Bus Service in Mid-sized Cities of India: A Case Study of Patna | Sinha et al., 2017 | India | Survey | To assesses the existing ‘quality of services’ of midi buses operating in Patna, India | Bus users were very much satisfied with the ticketing system, the condition of bus stops as the newly made bus stops have shelters of polycarbonate sheets and seating arrangements made of stainless steel. On the other hand, bus users were dissatisfied from the non-availability of the bus information at the bus stops, frequency of bus service and buses being on time. |
| 4 | Mumbai Urban Transport Project - A multi-dimensional approach to improve urban transport | Agarwal, 2013 | India | Report | To understand the lessons learned from implementation of the Mumbai Urban Transport Project (MUTP), a World Bank financed project | There was good economic returns. Increase in number of trains resulted in reduction in overcrowding, though, not to the extent expected at the time of conceptualizing the project mainly because the ridership has increased more than expected. |
| 5 | Influence of microscale environmental factors on perceived walk accessibility to metro stations | Bivina et al., 2019 | India | Survey | To explore the impact of various microscale built environmental factors on pedestrian satisfaction with regard to access to metro stations. | The government of Delhi exhibited a lackadaisical approach towards pedestrian satisfaction as evidenced by its lack of integrity with public transport services and lack of policies for pedestrian safety and security. |
| 6 | Results Of A Large-Scale Randomized Behavior Change Intervention On Road Safety In Kenya | Habyarimanaa & Jack, 2015 | Kenya | Randomized controlled trial | To test the efficacy of evocative messages, delivered on stickers placed inside Kenyan Matatus, or minibuses, in reducing road accidents. | There was a reduction in accidents, reduction in vehicle speed, improved safety, and increase in passenger empowerment. Text only messages were found ineffective, possibly due to limited literacy. |
| 7 | Road Traffic Injury Prevention Initiatives: A Systematic Review and Meta-summary Of Effectiveness in Low- and Middle-Income Countries | Staton et al., 2016 | LMICs | Systematic review | To identify road traffic injury (RTI) prevention initiatives tested in LMICs in the literature and perform a Meta-summary to determine their effectiveness at reducing crashes, injuries or fatalities | All the interventions showed to be effective in various degrees in reducing RTIs except for road improvement. Enforcement of legislation had an impact on the intervention success and sustainability. Educational interventions, which were commonly adopted due to low cost and low intervention complexity had limited effect. |
| 8 | Linking urban transport and land use in developing countries | Cervero, 2013b | Developing countries (Bogota, Ahmedabad and Curitiba) | Literature review | To know the impact of Bus Rapid Transit (BRT) and urban development in Bogota, Ahmedabad and Curitiba | The interventions helped in reducing congestion and improving urban infrastructure. Institutional barriers identified were; insufficiently trained staff, corruption, limited experience with urban planning, urban management, budgeting, finance and project supervision, institutional fragmentation. |
| 9 | Impact of public transport and non-motorized transport infrastructure on travel mode shares, energy, emissions and safety: Case of Indian cities | Tiwari et al., 2016 | India | Impact evaluation | We aim to estimate the likely range of the impacts of appropriate built environment and NMT and bus infrastructure on energy consumption and equivalent CO2 emissions in three medium size Indian cities (Udaipur, Rajkot & Vishakhapatnam) | Maximum reduction in CO2 emissions was achieved when both public transport and non-motorized transport infrastructures are improved |
| 10 | Urban transport and sustainable transport strategies: A case study of Karachi, Pakistan | Qureshi & Lu, 2007 | Pakistan | Review | To evaluate the existing transportation and infrastructure system, national transportation policies, and urban transportation projects and to determine if the current paradigm is moving toward or away from sustainable transportation. | Limited road space combined with poor maintenance, delayed repair work, bad quality construction, and absence of essential support functions created problems in satisfying the traffic demand. The negligence of successive governments in maintaining infrastructure, stations, and 23 level crossings that obstructed the circular railway trains from arriving on time were the main reasons for declining numbers of riders in Karachi Circular Railway. |
| 11 | Promoting low carbon transport in India: NMT Infrastructure in India: Investment, policy and design. | Tiwari & Jain, 2013 | India | Overview | The study aims to highlight gaps in implementation of policy, and identify appropriate policy and design interventions required to encourage NMT use in Indian cities. | Improved bicycle infrastructure, safer and walkable streets, reduced travel time, reduced speed of other vehicles in mixed lanes, reduced conflict between bicycles and other motorized vehicles resulted in increased speed of bicycles on the corridor. The risk imposed by buses came to 'zero' by 2009. |
| 12 | Transforming cities with transit: Transit and land-use integration for sustainable urban development | Suzuki et al., 2013 | India & Columbia | Evaluation report | This study identifies ways to promote more sustainable forms of urban growth that result in increasingly sustainable travel behavior in rapidly developing cities of the world. | Eight major barriers to transit and land-use integration were:  • lack of regional coordination at the metropolitan level  • sector silo behavior and practices at the city level  • inadequate policies and regulations for strategically creating “articulated densities” (densities that are strategically distributed across parts of a metropolitan area)  • restrictive national regulations and administrative constraints  • inconsistencies in the planning instruments and deficiencies in their implementation  • inadequate policies, regulations, and supporting mechanisms for redeveloping built-up areas, particularly brownfields or distressed and blighted districts  • neglected urban design at the neighborhood and street level  • Financial constraints. |
| 13 | Sustainable transport | Winter, 2016 | LMICs | Report | Impact of BRT in different cities | Yichang, China: Impact analysis surveys in September 2015 showed a range of impressive impacts, such as improvements in perceptions of public transport, a mode shift among BRT users of 20% from cars & taxis, improved security & civic pride, and a reduced bus-vehicle kilometers traveled due to more efficient operations. In African cities: Cape Town, Johannesburg, and Lagos have introduced BRT and busway corridors that have significantly improved the efficiency of public transport. In Mexico the most vulnerable pedestrian groups—children and the elderly were able to walk securely. |
| 14 | Walking and Cycling in Indian Cities: A Struggle for Reclaiming Road Edges | Datey et al., 2012 | India | Working paper | An overview of the Delhi, Pune and Ahmedabad BRT corridor is given in the paper | Increased use of bicycle, safety for pedestrians and bicyclists and accessibility for opportunities in Delhi. Whereas in Pune, many obstructions were found on the NMT corridor. where road widths were less |
| 15 | Sustainable urban transport | Pardo et al., 2012 | Mixed regions (China and Columbia) | Case study | The paper describes the case studies of China and Columbia regarding sustainable urban transport infrastructure | Improved access to different parts of the city in Guangzhou. The percentage of riders satisfied with public transport rose from 29 per cent in December 2009 (when there was no BRT) to 65 per cent by the following year. In Bogota there was improved safety for bicycle riders that increased use of bicycles. |
| 16 | Big Cities, Big Challenges: Sustainable Urban Transport across Major Middle East and North African Cities | Enzelberger & Kahramane, 2015 | MENA regions (Turkey and Morocco) | Case studies | This report highlights the main problems affecting urban transport in MENA region and the crucial elements for consideration in developing sustainable urban transport | In Istanbul, Metro-Bus and Marmaray reduce travel times significantly. In Morocco, there was better access and shift in mode of transport |

**Table 1b: Study characteristics- Transport policies and policy measures**

| Sl. no | Title | Author & year | Country | Study design | Study objective | Findings |
| --- | --- | --- | --- | --- | --- | --- |
| 1 | A global analysis of urban design types and road transport injury: an image processing study. | Thompson et al., 2020 | Global cities | Review | To determine whether city typologies are associated with variation in rates of road transport injury burden. | The study highlights the opportunity for reducing global burden of road transport injury by embracing urban designs that emphasize characteristics captured within high transit city types. |
| 2 | Commentary: Status of road safety in Asia | Wismans et al., 2015 | 24 Asian countries of which 20 were LMICs | Review | To assess the status of road safety in Asia | The challenges to improve road safety in developing countries are larger than in HICs due to inadequate infrastructure, unplanned urbanization taking place, lack of a legal regulatory framework and a strong increase in motorization. Reliable data is needed to determine intervention strategies. |
| 3 | Framing social inclusion as a benchmark for cycling-inclusive transport policy in Kisumu, Kenya | Alando & Scheiner, (2016) | Kenya | Qualitative content analysis | To find out the extent to which Kenya’s economic development blueprint is consistent with its transport policy | Results show that while the Kenya Vision 2030 focuses on economic growth, the government has prioritized the implementation of its projects, thus diminishing the fragile opportunity for cycling inclusion presented by the transport policy. Various policy recommendations have been made for cycling inclusive policies. |
| 4 | Transport Policies and Development | Berg et al., 2017 | LMICs | Review | The objective of this paper is to review the broader direct and indirect benefits and costs of transport investments and policies in developing countries. | Implementation of transport policies in developing countries faces a number of challenges such as funding for transport infrastructure investments, construction of infrastructure is particularly costly in developing countries, and inefficient management. |
| 5 | BRT in the Philippines: A Solution to Manila and Cebu Traffic Problems? | Boquet, Y (2019) | Philippines | Review | This paper examines the current situation of congestion, and its adverse effects, in the two main urban areas of the Philippines, Manila and Cebu, and the plans to implement BRT | Implementing a BRT will require roadwork, financing, and a change in the way the bus system operates. A re-evaluation of intermodality between the renovated bus system, the rail system and two iconic Filipino transport modes was needed. |
| 6 | Improving health through policies that promote active travel: A review of evidence to support integrated health impact assessment | de Nazelle A et al., 2011 | Region not specified | Review | To review available literature regarding health impacts from policies that encourage active travel in the context of developing health impact assessment (HIA) models to help decision-makers propose better solutions for healthy environments. | Policies that increase active travel are likely to generate large individual health benefits through increase in physical activity for active travelers. In addition, there will be reductions in air and noise pollution. |
| 7 | Active transport in Africa and beyond: towards a strategic  framework | Loo & Siiba, 2018 | 7 African countries (Ghana, Kenya, Malawi, Nigeria, Rwanda, South Africa and Uganda). | Review | To provide the direction towards how active transport (AT) policy development can be improved in Africa | Those countries (Kenya, Nigeria and S. Africa) which had specific AT policies did better (in terms of improved AT safety conditions) than those with general transport policies covering AT. |
| 8 | Transport and environment in developing countries: Comparing air pollution and traffic accidents as policy priorities | Vasconcellos, 1997 | Developing  countries | Review | NA | Developing countries need safer streets more than cleaner cars, and that a change in current priorities is required. |
| 9 | Road transport in Urban India: Its implications on health | Solanki et al., 2016 | Urban India | Review | The objective of this paper is to review the multiple impacts on health because of road transport in urban areas. | Harmful health effects described were road traffic accidents, air pollution, noise pollution, physical inactivity, and allergies. In Ahmedabad, after the introduction of BRT there has been more than 50% decrease in road traffic fatalities in the BRT corridor. |
| 10 | Road-Traffic Injuries: Confronting Disparities to Address a Global-Health Problem | Ameratunga et al., 2006 | LMICs | Review | To summarize the characteristics of the rise in road-traffic injuries and present an evidence-based approach to prevent road-traffic crashes. | Evidence based approaches identified in the review are as follows: Using safer transport & land use policies, safer roads, safer vehicles and safer people |
| 11 | Health and Road Transport in Pakistan | Hyder et al., 2006 | Pakistan | Systematic review | The specific objectives of this study were: (1) to review road transport policies in Pakistan and their documented impacts on health outcomes (2) to review implemented road-transport-specific interventions; and (3) to identify gaps in policy and implementation for future research in the road transport sector. | There has never been an approved transport policy in Pakistan. Research studies have emphasized the importance of the link between good transport policies and health in Pakistan in their discussion sections, but without suggested interventions. The greatest measured impact from road transport in Pakistan is the loss of life and disability caused by vehicular crashes |
| 12 | Transforming Our Cities: Best Practices Towards Clean Air and Active Transportation | Glazener & Khreis, 2019 | Not stated (but includes few LMICs) | Literature review | To review the literature and state the best practices for clean air and active transportation in urban areas | The best practices of clean air and active transportation have produced impressive results, which are improved when enacted simultaneously in integrated policy packages. |
| 13 | Social determinants of health Sectoral briefing- Series 3 | WHO, 2011a | Not stated but includes LMICs | Report | This briefing describes challenges faced by transport policy-makers and authorities, how they address them, and areas for potential collaboration between health and transport | It is important to increase the visibility of the safety and accessibility needs of marginalized groups and vulnerable road users, including those with disabilities.  Compact and mixed-use urban development’s save spaces for walking and cycling, good quality public transport reduces individual motorized travel, which in turn reduces the risk of road injuries and harmful emissions, and increases physical activity. |
| 14 | Transport and land-use policies in Delhi | Tiwari, 2003 | India | Bulletin by WHO (special theme- health impact assessment) | The review was done to understand the transport and land use policies in Delhi | Infrastructures for pedestrians should be created to ensure safe approaches to bus stops, and road usage for public transport vehicles. |
| 15 | Negotiating "Streets for All" in Urban Transport Planning: The Case for Pedestrians, Cyclists and Street Vendors in Nairobi, Kenya | Khayesi et al., 2010 | Kenya | Review | This paper uses the concept of “streets for all” as the analytical basis to comment on the neglect of pedestrians, cyclists and street vendors in transport policy and practice in the city of Nairobi. | Implementing a “streets for all” policy required dedicating financial and human resources to incorporate the needs of these street users in road design, land use planning, transport infrastructure investment, urban policy and decision-making, legislation and services. |
| 16 | GIS-Based Safety Bus Stops-Serdang and Seri Kembangan Case Study. [Subsection: Public Transport in Pakistan:  A Critical Overview] | Hazaymeh, K. (2009). | Pakistan | Review | To provide a critical overview of public transport policy in Pakistan. | This overview highlights the core problem of the continuing failure of cities in Pakistan to develop and manage their public transport systems. Several factors such as the importance of governance, capacity building, and urban planning were responsible for the failure. |
| 17 | Walkability and pedestrian facilities in Asian cities: state and issues. | Leather et al., 2011 | Asian countries | Review | The study provides information on the current pedestrian infrastructure in selected Asian cities | This review indicates the need to overhaul the existing pedestrian guidelines or develop appropriate guidelines for Asian cities. |
| 18 | Policies and programs for road safety in developing India | Mittal, 2008 | India | Review | This commentary reviews various aspects of road safety in heavily populated and developing India where motor vehicle trauma is a modern epidemic | The National Road Safety Council (NRSC) has tried to formulate and finalize a road safety policy for India, but without much fruitful results. The existing NRSC suffers from certain pitfalls and is not in a position to attain the stated objectives in coordinating the road safety activities |
| 19 | Non-Motorized Transport Policy in India: The need for a reform agenda | Kumar et al., 2015 | India | Report | NA | The ministry of urban development has encouraged Indian cities through initiatives and programmes to adopt NMT as a key component of their integrated urban transport system. These initiatives are forward thinking but have not yielded desired results. |
| 20 | Transport Strategy to Improve Accessibility in Developing Countries | Roberts & Babinard, 2004 | Developing countries | Report | This paper outlines guidance for addressing the access and mobility needs of disabled and elderly people in the context of the World Bank’s mission to reduce poverty and discusses the main challenges for providing inclusive transport. | Challenges to put policy into practice were inadequate monitoring and enforcement of compliance with existing accessibility legislation, and lack of resources for implementation. |
