# Supplementary material for: Contextual factors influencing the urban mobility infrastructure interventions and policies for older adults in low- and middle-income countries: a realist review
Source: BMC Public Health. 2022 Aug 5;22:1489. doi: 10.1186/s12889-022-13875-6 (PMC9354419; doi:10.1186/s12889-022-13875-6)
Supplement: Supplementary file 4 — Additional file 4: Table 2a. Urban transport interventions (public transport). Table 2b. Urban transport interventions (non-motorised transport). [file 12889_2022_13875_MOESM4_ESM.docx]

***Table 2a: Urban transport interventions (public transport)***

| Context | Mechanisms | Outcomes |
| --- | --- | --- |
| Introduction of BRT corridor in Mexico *(TransMilenio)*, African countries, India (*Janmarg)*, Columbia, China, Brazil and Morocco | Change in behavior of users seen by a shift from private motorised vehicle to public transport | - Reduced travel times, congestion and air pollution - Improved efficiency of public transport |
| Placing stickers with evocative messages (with or without images) inside the ‘*Matatus’ or ‘mini buses’* along with a radio campaign in Kenya | Older adults were empowered to complain about the rash driving, change in behavior of drivers was seen due to incentives given to keep the stickers in place inside the bus  Illiterate users were not able to read the stickers which did not have images | - Reduced accidents and improved safety when stickers had both text as well as images - Plain text stickers were not as effective as those text and images |
| Involving multiple bus agencies for providing services in China | Inter-agency cooperation and support from international agency in the project from the beginning | Successful implementation of BRT and cost efficient |
| *‘Boda-boda’* (motorcycle taxis) in Nigeria and South Africa along with the decision from government to expand cell phone networks | It helped older adults to book these taxis through a phone call without any difficulty | - Improved access to remote areas and medical care - Better opportunities for older adults to transport their farm produce to market |
| Public transport interventions in Turkey and India | Coordination between the government agencies (departments allocated with transportation projects), permissions from agencies involved, involvement of NGOs, funding from respective government departments, and coordination between local and state administration | Successful implementation of interventions |
| Mexico, India and sub-Saharan Africa | Coordination between the government agencies | Improved sustainability for accessible transportation |
| Rail transport and road transport projects at Mumbai to improve the capacity, efficiency and safety | Coordination between the government agencies | - Enhanced capacity of bus and rail systems. - Reduced congestion, travel time, emission from motorized transport and improved safety for users |
| Improving built environment, NMT and bus infrastructure in Indian cities (Udaipur, Rajkot and Vishakhapatnam) | Inter-sectoral coordination and good governance for project implementation | Better air quality when both public transport & NMT were simultaneously improved |
| Electronic ticket system, comfortable seating in bus, proper shelter in bus stations in India | As it was comfortable and acceptable to users, thus shifting the mode of transport | Increased ridership in bus |
| Shifting to new buses, off-board ticket collection and exclusive bus lanes in Mexico | Shift in mode of transport due to acceptable and accessible transport | Increased ridership in bus |
| *‘Marmaray metro bus’* project in Turkey | Accessible metro stations for vulnerable population | Reduced travel time |
| Introduction of new bus network to complement the tram system, improving bus shelters and timing information in bus station in Morocco | Good integration with other modes of transport resulting in better access and shift in mode of transport | Increased opportunities to travel independently |
| Legislations on seat belt, helmet and cell phone usage to improve road safety and higher penalties, increased police enforcement, public awareness/ education, speed control by placing rumble strips and construction of new roads in LMICs | Behavior changes among users and good governance | Reduced road traffic injuries and increased helmet use |
| Revitalizing Karachi circular railway (KCR) in Pakistan | There was a lack in the maintenance of infrastructure and coordination within the government agencies | Decline in the number of users and failure to function as per the plan |
| Urban bus scheme for infrastructure improvement of buses, construction of Lyari expressway and the northern bypass in Pakistan | Good governance and intersectoral coordination among agencies involved | Reduced congestion and crowding |
| BRT corridor in Pakistan | A lack of comprehensive plan in integrating the planning and implementation of the project by different agencies | Failure of the planned intervention |

***Table 2b: Urban transport interventions (non-motorised transport)***

| Context | Mechanisms | Outcomes |
| --- | --- | --- |
| *‘Complete street project’* in Mexico, that included widening sidewalks, redesigning of junctions, bicycle lanes and parking, recovery of public space and green space along the entire BRT corridor | Change in behavior by increase in the number of trips by foot | Increased walking for transport and recreation due to improved pedestrian accessibility and safety |
| The pedestrian program *‘Camina’* was taken up to improve Mexico city’s walk environment | Change in behavior by increase in the number of trips by foot | Vulnerable groups like older adults and children were benefitted |
| Improved access to BRT corridor by making better pedestrian pathways and bicycle lanes in Mexico, African countries, India, Columbia, China and Morocco | Change in behavior of users and shift in mode of transport | Improved access to public transport |
| Construction of walkways, sidewalks and plazas connecting to BRT in Bogota, Columbia | ‘Top-down’ approach was used where policy makers made policy and city administration implemented it | Safe access to public transport |
| Bike sharing system in China: those who used BRT could use the ticket to avail free bike ride in the first hour | A shift in the mode of transport to BRT | Improved NMT infrastructure, reduced air pollution, reduced travel time, increased safety and reduction in accidents |
| Improving pedestrian pathway and BRT in Nanded, Bengaluru and other cities | Poor design of bicycle lanes, obstructions on footpaths and bicycle lanes, use of interlocking blocks resulting in a bumpy ride and stagnation of water leading to slippery route, lack of parking space for bicycles, lack of security, unclean sidewalks, unsafe crossing facilities, lack of shaded sidewalks, street lights and police patrol | Poor uptake of NMT |
| Accessible and safe roads for pedestrians and bicyclists in Delhi, Pune and Ahmedabad | - Increase in number of trips by NMT modes in Delhi - In other cities, there was a lack of understanding of the need for NMT infrastructure, and gaps in planning and implementation of project | An increase in the use of bicycle and pedestrian safety was only seen in Delhi |

***Table 2c: Transportation policies and policy measures***

| **Outcome** | **Countries** | **Policies/ policy measures** |
| --- | --- | --- |
| Road safety measures | Global cities and LMICs (49, 50) | *Policy measure:* Investing in urban designs to shift the transport mode from motor vehicles to low-risk modes like rail or safe active transport. |
|  | Pakistan (51) | National Highway Safety Ordinance (2000) - a major breakthrough in road transport regulation |
|  | LMICs [Vietnam, Ghana, Columbia, Mexico, Nigeria, Peru, Argentina, Brazil, Ecuador, Philippines, Thailand, sub-Saharan Africa, Kenya & Uganda] (56) | *Policy measures:* Wearing motorcycle helmets rule (Vietnam, 2007), Rumbler strips to reduce speed (Ghana, 2000), Introduction of BRT (Columbia, Mexico, Brazil, and Nigeria), ‘Conduce Sin Alcohol’ (drive without alcohol) programme in Mexico, transit-oriented development scheme (Brazil, 1965). |
|  | 20 LMICs [Afghanistan, Bangladesh, Bhutan, Cambodia, People's Republic of China, Indonesia, India, Lao PDR, Malaysia, The Maldives, Mongolia, Myanmar, Nepal, the Philippines, Pakistan, the Russian Federation, Sri Lanka, Thailand, Timor-Leste and Vietnam] (52) | Global Plan for the Decade of Action for Road Safety (WHO 2011). The following are the five focus areas included in the plan   1. Road safety management 2. Safer roads & mobility 3. Safe vehicles 4. Safe road users 5. Post-crash response |
|  | India (46, 53) | Road safety policy for India (2003) and National Road Safety Policy was approved in India in 2010 |
|  | 7 African countries (Ghana, Kenya, Malawi, Nigeria, Rwanda, South Africa and Uganda) (60) | Following were policies for transport safety:  Transport Policy of Addis Ababa (Ethiopia, 2011)  Integrated National Transport Policy (Kenya, 2015)  Public Transport Policy and Strategy (Rwanda, 2012)  National Transport Master Plan (Uganda, 2009)  Draft National Transport Policy (Malawi, 2014)  Urban Transport Policy for Greater Maputo (Mozambique, 2006)  Draft National Non-motorized Transport Policy (South Africa, 2008)  Draft National Transport Policy (Zimbabwe, 2013)  Ghana National Transport Policy Integrated Transport Plan for Ghana (2008-2010)  The National Transport Master Plan of Liberia (2012)  Draft National Transport Policy (Nigeria, 2010)  Integrated Transport Policy Strategy and Investment Plan (Sierra Leone, 2013)  Integrated National Transport Strategy Study (Mauritius) |
| Thrust on access to healthcare, employment, and leisure | Kenya (54) | Kenya Vision 2030 & Integrated National Transport Policy (Kenya, 2015)- To develop and maintain an integrated, safe and efficient transport network |
|  | Kenya (56) | Bicycle Workshop, Cycle to School and Curriculum Development for Bicycle Mechanics projects in Kenya- these initiatives impacted on income generating activities and access to social services like healthcare |
|  | Uganda (56) | Cycle to School and Bicycle Ambulances projects – access to healthcare |
|  | Ecuador (56) | Metrobus-Q system was introduced in Ecuador to minimize private motor vehicle usage |
|  | South Africa (55) | Price incentives -better employment opportunities due to transport subsidies in South Africa (Cape town) |
|  | Developing countries (43) | Interventions proposed for more accessible transport system: 1) visual contrast, color coding clear/intuitive signs, 2) basic sidewalk and crossing design, hazard markings, minimize steps and other hazards, 3) Raised pedestrian crossings, raise boarding platforms or low-floor vehicles, general training of operational staff 4) user awareness, priority seating, additional training of operational staff, tactile surfaces 5) Elevators, illuminated and audible signals, wheelchair location, special transport services |
|  | Pakistan (48) | Public Transport in the National Transport Policy, 1991  Public Transport in the Prime Minister’s Public Transport Scheme, 1991  Public Transport in the National Conservation Strategy (Agenda 21), 1992  Public Transport through Community-Based Welfare Organization.  National Integrated Transport Policy, 1998  Transport Sector Development Initiative (TSDI), 1999  National Transport Strategy, 1999 |
| Policy measures for improved environmental conditions and well-being | Pakistan (51) | National conservation strategy (1992), focused on reducing air pollution, reducing vehicle emission, and promotion of energy-efficient transport systems |
|  | LMICs [Mexico, Nairobi, Columbia, India & China] (57) | Car free policies, vehicle technologies and integrated policy packages for cleaner environment and better health outcomes |
|  | LMICs [Vietnam, Ghana, Columbia, Mexico, Nigeria, Peru, Argentina, Brazil, Ecuador, Philippines, Thailand, sub-Saharan Africa, Kenya & Uganda] (56) | *Policy measures:* Phasing out of the leaded gasoline, the reduction of sulphur in diesel and the introduction of natural gas to reduce air pollution by National Emissions Inventory in Mexico  Introduction of Marikina bikeway system to reduce congestion and emissions in Philippines  Subsidies programme for vehicle owners to shift to cleaner fuels (natural gas) in Columbia, Peru, Argentina and Brazil.  Introduction of BRT to reduce noise pollution in Columbia. |
|  | India (58) | The Government of India made policies in 1997 for reducing air pollution and measures such as introduction of metro rail system and phasing out older buses was taken to reduce vehicular emission |
|  | Pakistan (48) | The People’s Train and Awami (People’s) Bus Train Projects |
|  | LMICs (55) | Policy measures: Regulations to reduce emissions and infrastructure investments such as building new transport infrastructure, upgrading existing links and technology, and improving transport services. |
|  | Philippines (45) | Policy measure: BRT implementation for accessible transport and cleaner environment |
|  | LMICs (59) | Policy measure: Active travel measures and behaviors such as promotional campaigns and change in physical infrastructure. |
|  | Developing countries (62) | Policy measures: congestion relief, vehicle emission control, re-organization of traffic in a given street, the use of alternative routes, contraflow bus lane and bus corridor resulted in reduced emissions |
|  | India (46) | National urban transport policy, 2006 - Provide cleaner, efficient, & alternate mode of transport to ensure safe, affordable, quick, comfortable, reliable and sustainable transportation systems in the cities for mobility needs of the residents. |
| Measures to improve NMT | 1. Kenya (61) 2. Malaysia (63) 3. Philippines (63) 4. Singapore (63) 5. Bangladesh (63) 6. Bhutan (63) 7. Mongolia (63) 8. India (47, 63) 9. Sri Lanka (63) 10. Indonesia (63) | 1. Sub-Saharan Africa Transport Policy Program “Streets for all”  2. The Tenth Malaysia Plan (2011-2015) – focus on “public transport as the primary spine, supported by a pedestrian-friendly street network.”  3. The draft strategy states that: “Reserving and reclaiming space for pedestrian traffic is as important as providing lanes for cars.”  4. Providing pedestrian walkways, pedestrian crossings, traffic signs and interchanges  5. National transport policy gives emphasis on pedestrians  6. Tenth five-year plan (2008-2013) - encourage NMT such as walking and cycling  7. National transport strategy - improved facilities for pedestrians  8. National Urban Transport Policy (2006) encourages integrated land use and transport planning, public transport and non-motorized modes by giving them priority in investments.  9. National Transport Policy (1991) - encourage the use of public transport, high occupancy vehicles and non-motorized transport.  10. Traffic and Road Transport Act of Indonesia (Act 22/2009) |
